# *In vitro* and *in vivo* evaluations of glass-ionomer cement containing chlorhexidine for Atraumatic Restorative Treatment

**DOI:** 10.1590/1678-7757-2016-0195

**Published:** 2017

**Authors:** Cristiane Duque, Kelly Limi Aida, Jesse Augusto Pereira, Gláucia Schuindt Teixeira, Angela Scarparo Caldo-Teixeira, Luciana Rodrigues Perrone, Karina Sampaio Caiaffa, Thais de Cássia Negrini, Aline Rogéria Freire de Castilho, Carlos Alberto de Souza Costa

**Affiliations:** 1 Univ. Estadual Paulista (UNESP), Faculdade de Odontologia de Araçatuba, Departamento de Odontologia Infantil e Social, Araçatuba, SP, Brasil; 2 Universidade Federal Fluminense (UFF), Instituto de Saúde de Nova Friburgo, Departamento de Odontologia, Nova Friburgo, RJ, Brasil; 3 Universidade Federal do Rio Grande do Sul (UFRGS), Faculdade de Odontologia, Departamento de Odontologia Conservadora, Porto Alegre, RS, Brasil; 4 Universidade Estadual de Campinas (UNICAMP), Faculdade de Odontologia de Piracicaba, Departamento de Odontologia Infantil, Piracicaba, SP, Brasil; 5 Univ. Estadual Paulista (UNESP), Faculdade de Odontologia de Araraquara, Departamento de Fisiologia e Patologia, Araraquara, SP, Brasil

**Keywords:** Dental atraumatic restorative treatment, Chlorhexidine, Glass ionomer cements

## Abstract

**Objectives:**

Addition of chlorhexidine has enhanced the antimicrobial effect of glass ionomer cement (GIC) indicated to Atraumatic Restorative Treatment (ART); however, the impact of this mixture on the properties of these materials and on the longevity of restorations must be investigated. The aim of this study was to evaluate the effects of incorporating chlorhexidine (CHX) in the *in vitro* biological and chemical-mechanical properties of GIC and *in vivo* clinical/ microbiological follow-up of the ART with GIC containing or not CHX.

**Material and Methods:**

For *in vitro* studies, groups were divided into GIC, GIC with 1.25% CHX, and GIC with 2.5% CHX. Antimicrobial activity of GIC was analyzed using agar diffusion and anti-biofilm assays. Cytotoxic effects, compressive tensile strength, microhardness and fluoride (F) release were also evaluated. A randomized controlled trial was conducted on 36 children that received ART either with GIC or GIC with CHX. Saliva and biofilm were collected for *mutans streptococci* (MS) counts and the survival rate of restorations was checked after 7 days, 3 months and one year after ART. ANOVA/Tukey or Kruskal-Wallis/ Mann-Whitney tests were performed *for in vitro* tests and *in vivo* microbiological analysis. The Kaplan-Meier method and Log rank tests were applied to estimate survival percentages of restorations (p<0.05).

**Results:**

Incorporation of 1.25% and 2.5% CHX improved the antimicrobial/anti-biofilm activity of GIC, without affecting F release and mechanical characteristics, but 2.5% CHX was cytotoxic. Survival rate of restorations using GIC with 1.25% CHX was similar to GIC. A significant reduction of MS levels was observed for KM+CHX group in children saliva and biofilm 7 days after treatment.

**Conclusions:**

The incorporation of 1.25% CHX increased the *in vitro* antimicrobial activity, without changing chemical-mechanical properties of GIC and odontoblast-like cell viability. This combination improved the *in vivo* short-term microbiological effect without affecting clinical performance of ART restorations.

## Introduction

Early childhood caries (ECC), mainly in developing countries, is the most prevalent chronic disease in childhood and, consequently, a pending public health problem^6^. Depending on the severity of ECC and the number of dental sources of infection, this disease causes functional, aesthetic and psychosocial disorders that reduce the quality of life of children and their families^6^. The decay process of ECC generally tends to advance and become more difficult and costly to repair the longer it remains untreated. An alternative for the treatment of ECC is the Atraumatic Restorative Treatment (ART). ART is a definitive restorative treatment which consists of removing demineralized tooth tissues using minimal intervention to preserve the tooth structure and restoring the dental cavity with glass ionomer cement (GIC)^9^. The correct execution of ART procedures may change the balance of the oral microbiota, reducing cariogenic microorganisms^7^. This factor is relevant, because children affected by ECC have high counts of cariogenic bacteria in saliva, such as mutans streptococci and lactobacilli, and other species such as *Candida albicans* ^3^. Additionally, residual microorganisms can be found in dentin after partial caries removal procedures with ART. Some researchers have suggested the incorporation of antimicrobial agents into glass ionomer cements^4^ ^,^ ^5^ ^,^ ^27^. Chlorhexidine (CHX) presents a wide spectrum of activity against Gram positive bacteria, especially mutans streptococci, Gram negative, aerobic and facultative anaerobic bacteria, and fungi^8^. Studies have suggested that the incorporation of chlorhexidine salts into glass ionomer cements (GIC) increases their antimicrobial activity without compromising their physical-chemical properties^11^ ^,^ ^25^ ^,^ ^26^. On the other hand, other studies have shown that the inclusion of chlorhexidine into glass ionomer cements promoted significant antimicrobial activity; however, this change induced negative effects on the biocompatibility and mechanical properties of the restorative material^13^. One clinical study evaluated the long-term outcome of ART using glass ionomer cement containing CHX^15^. Therefore, the objectives of this study were 1) to evaluate the *in vitro* influence of incorporating different concentrations of CHX on biological and physical-chemical properties of a GIC and 2) to investigate *in vivo* clinical/microbiological follow-up of the ART with GIC containing CHX.

## Material and methods

### Dental materials

GIC used was Ketac Molar Easy Mix^®^ (KM, 3M ESPE, Seefeld, Bavaria, Germany). This material was modified by adding 1.25 and 2.5% chlorhexidine digluconate (CHX - C9394 Sigma-Aldrich, Steinheim, Westphalia, Germany) without altering liquid/powder ratio, as proposed by Türkün, et al.^26^ (2008).

### *In vitro* study

#### Antimicrobial activity *Microorganisms and growth conditions*

*Streptococcus mutans* (ATCC 25175), *Lactobacillus acidophilus* (ATCC#IAL-523) and *Candida albicans* (ATCC 40176) were obtained from Oswaldo Cruz Foundation (FIOCRUZ, Rio de Janeiro, RJ, Brazil). *S. mutans* and *L. acidophilus* were cultured on Mitis Salivarius Agar (Difco Laboratories, Detroit, MI, USA) with 0.2 UI bacitracin and Rogosa Agar (Difco Laboratories, Detroit, MI, USA) for 24-48 h at 37°C in 5% CO2. *Candida albicans* were grown in Sabouraud Dextrose Agar (Difco Laboratories, Detroit, MI, USA) for 24-48 h at 37°C in aerobic conditions. Subsequently, colonies were transferred to Brain-Heart Infusion broth (BHI; Difco Laboratories, Detroit, MI, USA) for 18-24 h at the same conditions. Cultures were adjusted to 1-5x10^8^ cells/mL in order to obtain an inoculum for subsequent tests.

#### Agar diffusion test

This test was conducted according to Castilho, et al.^5^ (2012). Twelve 5-mm-diameter wells were made in BHI agar plates and completely filled with KM, KM with 1.25% CHX, and KM with 2.5% CHX. All materials were handled under aseptic conditions according to the manufacturer's instructions, inserted into wells using a syringe (Centrix Inc., Shelton, CT, USA) and light activated (Blue Star III - Microdont, Sao Paulo, SP, Brazil) for 30 s. Light output was periodically checked (approximately 500 W/cm2). Positive control used was 0.2% CHX. After 2 h of material diffusion, the plates were incubated for 24 h in each microorganism's conditions. Then, inhibition zones around the materials were measured using a digital caliper.

#### Biofilm assays

This biofilm assay was based on a methodology previous described by Hu, et al.^12^ (2013). Five cylindrical of each KM group containing or not CHX were prepared using cylindrical molds (2 mm thick and 4 mm diameter) and individually suspended in 24-well plates (Corning Inc., New York, NY, USA) containing 2 mL of BHI broth supplemented with 1% sucrose and 2 µl of inoculum. The plates were incubated in 5% CO_2_ at 37°C for 24 h. After this period, GIC samples were washed, immerged in 500μl of 0.9% NaCl solution and sonicated in an ultrasonic cell disruptor at 7 W for 30 s (Branson, Sonifier 50, Danbury, CT, USA). This solution was diluted and plated on BHI agar and incubated for 48 h at 37°C. Then, bacterial colonies were counted and expressed in colonies forming units/ mL (CFU/mL). Three independent assays (n=15) were performed for the analysis.

#### Cytotoxicity assays

These assays were conducted in accordance with Castilho, et al.^5^(2012). Briefly, MDPC-23 odontoblast-like cells were used. The cells were seeded (30,000 cells/cm^2^/well) in sterile 24-well plates and maintained for 24 h in an humidified incubator at 5% C0_2_ and 95% air at 37°C (Isotemp; Fisher Scientific, Pittsburgh, PA, USA). Ten round-shaped samples of each group (2x4 mm) were prepared in stainless-steel molds, light-cured for 30 s and maintained for 1 h at 37°C in relative humidity. The specimens were then inserted into sterile 24-well plates containing DMEM (Gibco, Fisher Scientific, Hampton, NH, USA) for 24 h. After that, 800 µL of the extract from each well was applied to previously cultured MDPC-23 cells for 24 h. Cell metabolism was analyzed using methyl tetrazolium (MTT) assays. The means were calculated for the groups and transformed into percentages, and negative control was defined as having 100% cell metabolism.

### Measurement of mechanical properties

#### Compressive tensile strength and microhardness tests^5^

Ten specimens from each group were prepared in cylindrical molds for compressive strength (4x2 mm) and surface microhardness tests (3x6 mm). Compressive tensile strength tests were performed in an Instron universal test machine (4411, Instron Co., Canton, MA, USA) in a vertical position using a load at a crosshead speed of 1.0 mm/min until failure occurred and the values were calculated by dividing the load (F) by the cross-sectional area and converted to MPa. Microhardness was measured using a microhardness tester (Shimadzu HMV-2000 Micro Hardness Tester; Shimadzu Corporation, Kyoto, Keihanshin, Japan), under a static load (Knoop) of 50 gf for 5 s. Five indentations were randomly performed, 500 um apart, on the top surface of the material and hardness means were obtained for each sample.

### Measurement of chemical properties

#### Fluoride release^23^

Six specimens of each group were made with 5 mm and 2 mm diameter, with a surface area of 0.71 cm^2^. Each specimen was placed in 4 ml of deionized water under agitation at room temperature for 24 h. An equal volume of TISAB II (acetate buffer 1.0 M, pH 5.0, containing NaCl 1.0 M and 1,2-cyclohexanediaminetetraacetic 0.4%) was added to the tubes. The specimens were daily washed with deionized water, dried with absorbent paper and transferred to new tubes containing 4 ml of deionized water. The solutions from 24 h and 7 days were collected and stored at 4°C for the released fluoride using a fluoride-specific electrode (Orion 9609-BN, Orion Research, Inc., Beverly, MA, USA) connected to a digital ion-analyzer (Orion 720A, Orion 9609-BN, Orion Research, Inc., Beverly, MA, USA), previously calibrated with standard solutions of 0.0625 to 1 or 1 to 16 mg F/ml in TISAB II, and expressed in mg F/cm^2^.

### *In vivo* study

#### Study design

The present study was designed as a randomized controlled clinical trial with parallel groups. One hundred and tirty six three to six-year-old children from four public primary schools of Nova Friburgo (Rio de Janeiro, Brazil) whose parents signed a written consent were examined for dental caries status using the criteria developed by the WHO. Inclusion criteria were (1) good general health; (2) cooperative behavior; (3) at least one cavitated dentin carious lesion (occlusal or occluso-proximal cavities) in primary molars that had an opening wide enough for the smallest ART excavator access - 0.9 mm diameter. Exclusion criteria were children with mixed dentition, teeth with pulpal exposure, fistula, abscess or dental mobility, and history of sensitivity and/or spontaneously pain. The study was approved by Research Ethics Committee of the Federal Fluminense University (reference number 056/2010) and registered at the Clinical Trials (Identifier: NCT02459730). Parents and/or caretakers were informed in writing about the investigation and treatments. Children whose parents or caretakers filled in and signed the consent forms were included in the study. Sample size calculation was based on failure rates reported for conventional approximal ART restorations using high-viscosity glass ionomer cements (HVGIC) in primary posterior teeth (29%) after one year^1^. For ART restorations with HVGIC containing CHX, there were no reliable failure rate data. It was considered a positive outcome if the results were similar to those with HVGIC, showing clinical equivalence. A hypothetical minimal difference of 20% among groups were considered with a probability of type I error of 5% and a power of 80%. A minimum of 41 restorations were calculated *per* group. However, considering lost follow-up, the final sample was increased in 20% resulting in at least 49 restorations *per* group.

#### ART procedures

An independent dentist randomly distributed children in two groups. ART restorations and clinical evaluation were performed by a trained and previously calibrated pediatric dentist (CD), aided by two trained graduate students (LRP and KSC), using a portable bed and an operating light. The mean kappa value for the intra-examiner reproducibility was 0.78. Restorations were performed according to the ART approach described by Frencken, Taifour and van't Hof^9^ (2006). Briefly, carious lesions were prepared by removing infected dentin with hand instruments. No local anesthesia was used. Relative moisture isolation was performed with cotton wool rolls. Then, the cavities were conditioned with liquid from the material, washed and dried with cotton pellets. They were subsequently filled using the press finger technique with one of the randomly selected materials: (1) Ketac Molar Easy Mix® containing 1.25% CHX (KM+CHX; n=17 children; 49 restorations), or (2) Ketac Molar Easy Mix® as a control group (KM; n=19 children; 68 restorations). Each tooth was considered as the sampling unit. However, all carious teeth indicated to ART in each child were treated exclusively with one of the materials tested. After the removal of material excess and adjustment of the occlusion using the carver instrument, the restoration was coated with a layer of petroleum jelly. Multiple-surface cavities were filled after the placement of plastic bands and wedges. The dentist gave instructions to caregivers for children not to eat solid food for one hour.

### Follow up

An independent dentist, previously trained and calibrated, evaluated the restorations after 7 days, 3 months and 1 year of treatment. Following the ART criteria adopted for approximal restorations, as proposed by Roeleveld, et al.^24^ (2006), restorations were considered as a success (codes 00 and 10), failure (codes 11-40) or unavailable (codes 50-91). All carious teeth were treated with the same GIC used in molars for each patient, but only molars were considered for statistical analysis. New restorations were carried out to replace failed restorations but they were not considered in subsequent analysis. Children were encouraged and instructed on dental hygiene, and received all other necessary oral care.

### Microbiological assays

Unstimulated whole saliva and pooled supragingival biofilm samples from all buccal and lingual smooth surfaces, except from the interior of the cavities, were collected from each subject. A sterile plastic disposable (Greiner, Frickenhausen, Germany) was used to collect the biofilm^22^. Biofilm samples were placed immediately into 1 mL microtubes containing Tris-EDTA buffer (10 mM Tris-Hcl, 0.1 mM EDTA, pH 7.5). Collections were performed at least 1 h after feeding and the tubes were transported on ice and processed within 2 h. The samples were homogenized and the suspensions were serially diluted in 0.9% NaCl solution. Each dilution was cultivated in triplicate on the surface of Mitis Salivarius Agar (Difco Laboratories, Detroit, MI, USA) with sucrose and 0.2 U/ml bacitracin for isolation of mutans streptococci (MS). All plates were incubated at 37°C for 48 h in 5% CO_2_ atmosphere. After 48 h of incubation, the number of CFU was counted using a stereoscopic microscope and the results were expressed as CFU/mL

### Statistical analysis

Data were submitted to normality and homogeneity of variance tests, using the SPSS (version 17) statistical software. Anti-biofilm effects of KM groups were analyzed by Kruskal-Wallis and Mann-Whitney tests. ANOVA and Tukey tests were used to evaluate data from agar diffusion tests, cytotoxicity, mechanical properties and cumulative fluoride release assays. Kruskal/Wallis and Mann-Whitney tests were used to compare differences among material groups in the same period of time (7 days, 3 months or 1 year of evaluation) for microbiological analysis. The Wilcoxon test was used to compare microbiological differences within each material group considering each period evaluated. The Kaplan-Meier method and Logrank tests were applied to estimate survival percentages of restorations^2^. All statistical tests were considered with a 5% level of significance.

## Results

### *In vitro* study

#### Antimicrobial activity

**Figure 1:**
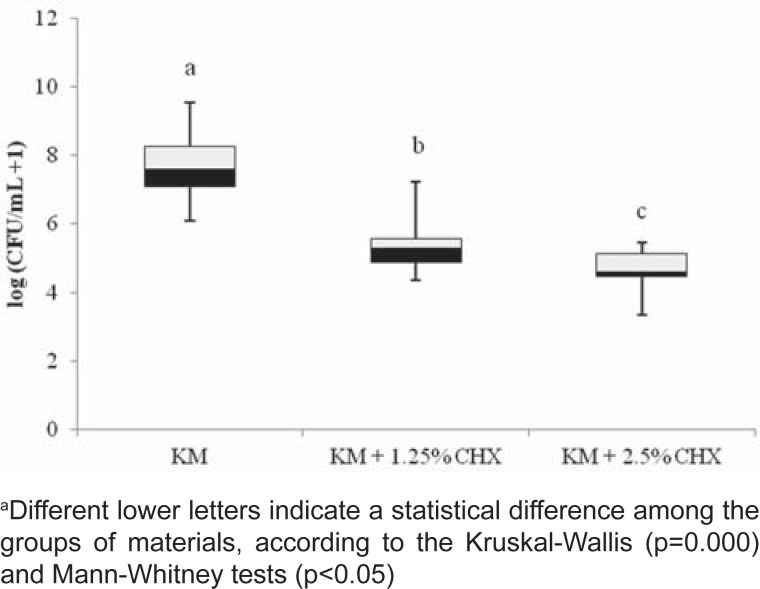
Box-whisker plots of the *S. mutans* anti-biofilm activity of the glass ionomer cements. Bars indicate minimum and maximum values. Black and white boxes indicate lower and upper quartiles, respectively. The line in the middle of the boxes is the median

Mean values of the results for the agar diffusion test are shown in Table 1. KM was not effective against all microorganisms tested. When CHX was incorporated into KM, it presented an inhibitory activity on all microorganisms. However, an increased CHX concentration did not significantly improve the antimicrobial effect. Regarding the S. *mutans* anti-biofilm activity, a statistical difference was observed comparing KM with and without CHX. The anti-biofilm action of KM+2.5% CHX was statistically better (p=0.007) than the observed for KM+1.25% CHX (Figure 1).

**Table 1:** Means ± standard deviations of inhibition zones (mm) for the glass ionomer cements against the tested microorganisms, using agar diffusion tests

	KM	KM+1.25% CHX	KM+2.5% CHX
*Streptococcus mutans*	0^a^	13.05±1.13^b^	13.87±1.12^b^
*Lactobacillus acidophilus*	12:00 AM	14.14±1.35^b^	14.59±0.80^b^
*Candida albicans*	12:00 AM	8.68±0.61^b^	9.18±1.61^b^

a Different lower letters indicate a statistical difference among the groups of materials, according to the ANOVA and Tukey tests (p<0.05)

#### Toxicity on odontoblast-like cells

**Figure 2:**
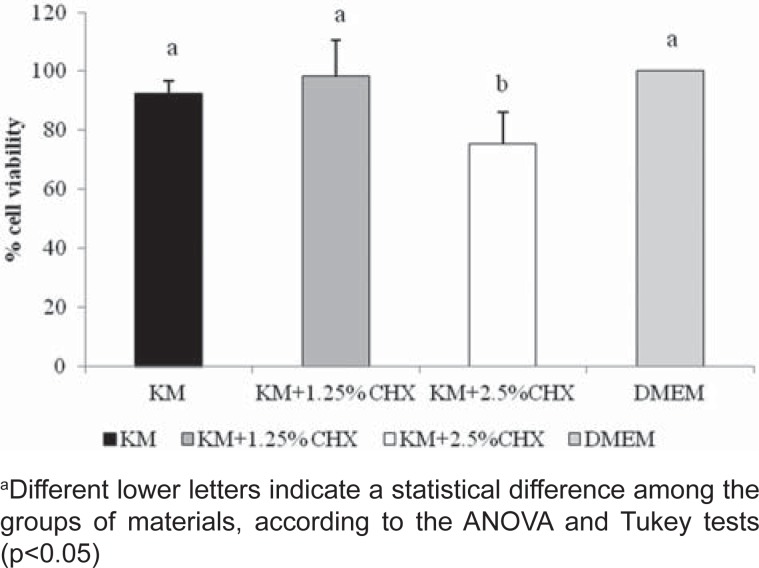
Means (standard deviations) of the percentage of odontoblast-like cell viability after exposure to extracts obtained from glass ionomer cements (MTT assays)

Figure 2 shows that KM and KM+1.25% CHX did not present a cytotoxic effect. However, when KM was associated with 2.5% CHX, a significant decrease in cell viability was observed.

#### Mechanical and fluoride release properties

**Figure 3:**
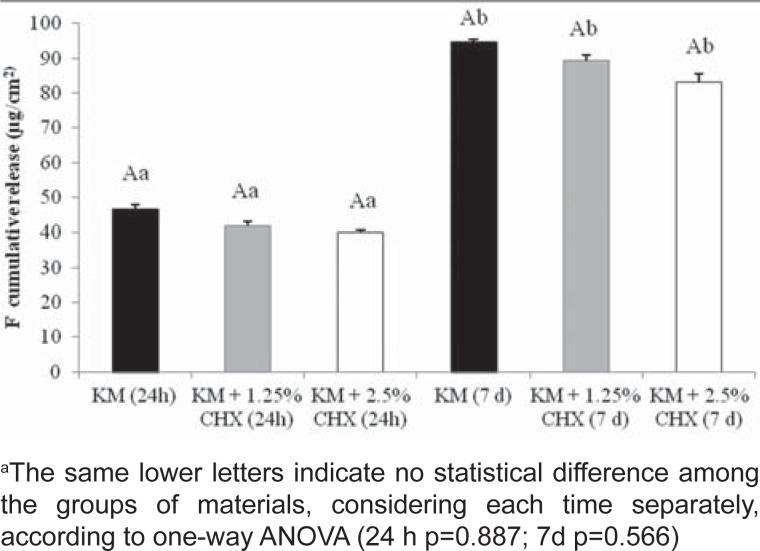
Means (standard deviations) of cumulative fluoride release (ugF/cm^2^) from glass ionomer cements containing or not containing chlorhexidine after 24 h and 7 days in deionized water

The results of compressive strength and microhardness tests are shown in Table 2 and the cumulative fluoride release is shown in Figure 3. CHX, in both concentrations, did not affect these properties when compared to control group.

**Table 2:** Mechanical properties (means ± standard deviations) of glass ionomer cements containing or not chlorhexidine

	KM	KM+1.25% CHX	KM+2.5% CHX	p value
Compressive strength (MPa)	36.32±12.56^a^	36.04±10.68^a^	36.64±15.18^a^	0.992
Knoop microhardness (KHN)	45.34±1.52^a^	45.02±1.09^a^	45.34±1.74^a^	0.908

a The same lower letters indicate no statistical difference among the groups of materials, according ANOVA (p>0.05)

### *In vivo* microbiological and clinical assessments

**Figure 4:**
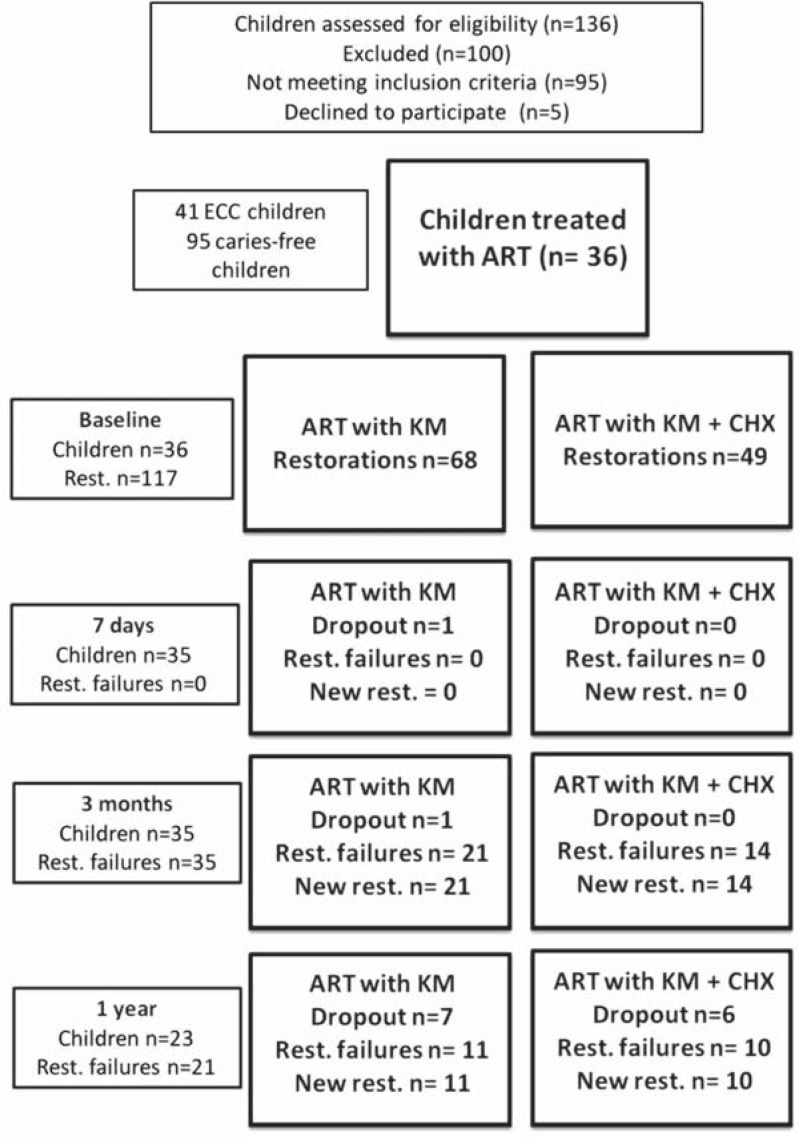
CONSORT flowchart of the patients and teeth treated with ART along this study

A CONSORT flowchart of the patients and restorations made along this study is described in Figure 4. The final sample consisted of 36 children, 20 (55.6%) of them were females. The population's mean age was 46.09±7.9 months. There was no statistical difference among groups of materials in relation to age (KM - 43.89±11.21; KM+CHX - 50.24±9.16), gender (KM: 11 females - 57.89%; KM+CHX: 9 females - 53%), mean ± standard deviation of molar surfaces treated (KM: 3.47±3.76; KM+CHX: 2.41±2.42) and number of teeth with single surface restorations (KM: 13 teeth - 19.11%; KM+CHX: 11 teeth - 22.44%) (p>0.05, ANOVA and Chi-square tests). Dmfs (decay, missing and filling surface) was 13.11 and 7.82 for KM and KM+CHX groups, respectively. In relation to molar restoration retention at different follow-up times, there were 21 failures in KM after 3 months and 11 after one year. For the KM+CHX group, failures were observed only in the third month (n=14) and one year after ART (n=10). However, survival percentage of restorations were similar among groups (Table 3). The main reason for restoration failures was partial or total fracture of restorations. Only two teeth treated with KM had secondary caries and one tooth treated with KM+CHX presented pulp inflammation after one year of ART. Microbiological analysis at follow-up times is presented in Table 4. The best antimicrobial performance was observed in the experimental group (KM+CHX) at the 7^th^ day follow-up for both saliva and biofilm analysis (p<0.05). For biofilm, MS reduction was also observed after 1 year of ART.

**Table 3:** Cumulative survival (means - %) and standard error of the means (SE) of ART restorations in primary molars treated with glass ionomer cements containing or not chlorhexidine

Intervals of time	N child *	N restorations	N dropout	N failed	Survival % Means (SE)
KM					
0 – 7 d	19	68	1	0	100 (0)
7 d – 3 m	18	67	1	21	68.19 (15.29)
3 m – 1 y	12	45	7	11	48.45 (8.36)**
KM+CHX					
0 – 7 d	17	49	0	0	100 (0)
7 d – 3 m	17	49	0	14	71.43 (14.57)
3 m – 1 y	11	35	6	10	48.57 (11.43)

* Nchild - number of children at start of interval, N restorations number of restorations at start of interval, N dropout number of restorations dropout at the end of interval, N failed number of restorations that failed at end of interval

** “Log rank: Chi-square= 0.022, df =1, p=0.882

**Table 4:** Median (Means) Standard Error of Mean of *mutans streptococci* counts (log_10_(CFU+1)) before (baseline) and after ART

	Saliva			Biofilm		
	KM	KM+CHX	p value	KM	KM+CHX	p value
Baseline	5.61 (5.50) 0.19^A^ ^a^	5.13 (5.27) 0.21^A^ ^a^	0.288	5.47 (5.20) 0.25^A^ ^a^	5.44 (5.24) 0.29^A^ ^a^	0.908
7 days	5.53 (5.47) 0.27^A^ ^a^	4.52 (4.48) 0.13^Bb^	0.012	5.67 (5.55) 0.23^A^ ^a^	4.59 (4.49) 0.22^Bb^	0.015
3 months	5.38 (4.98) 0.28^A^ ^a^	5.34 (5.38) 0.20^A^ ^a^	0.631	4.52 (4.79) 0.31^A^ ^a^	4.72 (4.83) 0.17^A^ ^a^	0.748
1 year	5.51 (5.54) 0.30^A^ ^a^	4.44 (4.68) 0.29^A^ ^a^	0.109	4.66 (5.11) 0.55^A^ ^a^	4.25 (4.59) 0.29^Ab^	0.361

A Different capital letters indicate a statistical difference among the materials (KM x KM+CHX), considering saliva and biofilm separately, according to Kruskal-Wallis and Mann-Whitney tests (p<0.05)

a Different lower letters indicate a statistical difference among the groups in each period of evaluation, according to Wilcoxon tests (p<0.05). For KM: 7 days x 3 months, p=0.034. For KM_+_CHX (saliva): Baseline x 7 days p=0.044; 7 days x 3 months =0.005; KM_+_CHX (biofilm): Baseline x 7 days p=0.050; Baseline x 1 year p=0.043

## Discussion

Several attempts have been made to introduce antimicrobial properties to restorative materials, including the incorporation of CHX salts into GICs, focusing on a new perspective for arresting residual caries after ART. Some authors demonstrated that the addition of CHX to glass ionomer cements improved the inhibitory effect against oral microorganisms, including *Streptococcus* and *Lactobacillus* species^7^ ^,^ ^19^ ^,^ ^20^ ^,^ ^25^. However, there are several differences in the methodologies used in these studies, mainly in the glass ionomer cement and chlorhexidine salt chosen for the experiments. The present study evaluated the inhibitory effect of adding chlorhexidine digluconate to Ketac Molar Easy Mix (KM) against *S. mutans*, *L*. *acidophilus* and *C. albicans*. Studies affirmed that digluconate and diacetate forms of chlorhexidine presented antimicrobial activity^7^ ^,^ ^20^ ^,^ ^25^ ^–^ ^27^. However, differences among them were found considering the inhibition zones against *S. mutans* and *L. acidophilus*, indicating that the type of salt may affect the antimicrobial action of CHX when associated with GICs^25^ ^–^ ^27^. The results of the current study are in agreement with the microbiological results obtained by Marti, et al.^20^ (2014) on *S. mutans* and *L. acidophilus*. Regarding *Candida albicans*, the current results differed from those obtained by Türkün, et al.^26^ (2008). This may be related to the glass ionomer cement chosen for the study and the agar diffusion methodology.

Additionally, this study evaluated the interference of CHX-modified glass ionomer cement on the formation of biofilm. *Streptococcus mutans* has been implicated as the main etiological agent of dental caries and plays an important role in dental biofilm formation^17^. This study demonstrated that the activity of GIC containing CHX against *S. mutans* biofilm was significantly higher when compared to the control group. Almost all studies in the literature demonstrated the antibacterial effectiveness of incorporating CHX to a conventional GIC by using the agar diffusion test and not by anti-biofilm activity^25^ ^–^ ^27^. In the present study, considering the limitations of the *in vitro* anti-biofilm assay, the incorporation of CHX significantly reduced *S. mutans* counts adhered to the GIC surface, with this anti-biofilm property increasing with the CHX concentration. Although this study used CHX digluconate and a different glass ionomer cement, the results are in accordance with those obtained by Hu, et al.^12^ (2013) and Du, et al.^10^ (2012). It was speculated that CHX released from the material could persist in the environment, due to its substantivity, creating a bacteriostatic effect and interfering on bacterial colonization and biofilm formation^17^.

Studies have demonstrated that the addition of antibacterial agents can change the mechanical properties of glass ionomer cements^20^ ^,^ ^25^ ^–^ ^27^. In the present study, the mechanical properties of GIC were not negatively affected by the addition of CHX (1.25 or 2.5%) when compared with the control group. These findings are consistent with those obtained by Takahashi, et al.^25^ (2006) and Hu, et al.^12^ (2013). According to Jedrychowski, Caputo and Kerper^13^ (1983), glass ionomer cement deteriorates after the addition of CHX at concentrations above 5%.

Fluoride is widely used as a highly effective anti-caries agent. Fluoride has also an antimicrobial activity, affecting bacterial metabolism, directly as an enzyme inhibitor or by reducing the acid tolerance of the bacteria^19^. Thus, fluoride release by glass ionomer cements is one of its most important properties, and is intrinsically associated with the anti-caries effect of the cement. In this study, fluoride release was not affected by the incorporation of both concentrations of CHX, similarly to Tüzüner, et al.^27^ (2011). Fluoride release of GICs after the addition of 10% CHX decreased over time, but remained measurable after 60 days^11^. It was speculated that it is an interaction between the fluoride ion and the cationic CHX molecule, resulting in the precipitation of salts with lower solubility, leaving less available fluoride^11^.

Biocompatibility is a property required for GICs, since these materials are usually applied in deep dentin and could release toxic components, which might indirectly affect the dental pulp^5^ ^,^ ^18^. High concentrations of CHX have cytotoxic effects on odontoblastic cells^18^. However, those results are related to *in vitro* direct contact of CHX with cells. In this study, toxicity against odontoblastic cells was observed only for the combination of GIC with the highest concentration of CHX (2.5%). The current results are in agreement with those obtained by Castilho, et al.^5^ (2012).

Regarding the present clinical trial, the survival rate after one year was approximately 48% for both materials. The majority of multiple-surface restorations for both groups (KM: 80.89% and KM+CHX: 77.56%) could explain partially the lower survival rate values. This result was slightly higher than 44.8% obtained by Kemoli, et al.^15^ (2009) and lower than 65% obtained by Yu, et al.^30^ (1998), using the same GIC in class II restorations, over a comparable period of time. Higher percentages were obtained in some studies presented in the systematic review of Amorim, et al.^1^ (2012). The authors found a weighted mean score of 71% for survival rate of multiple-surface restorations in primary teeth after one year. More recently, a cumulative survival rate of 80.9% was obtained for multiple-surface restorations using high viscosity glass ionomer cement within the same period of time^10^. The literature presents survival rates of ART restorations with high viscosity GIC in posterior teeth ranging from 74 to 100% and 31 to 100% for single or multiple surface, respectively, in the first year of evaluation^1^. This wide range of survival percentages observed in the studies, mainly for approximal surfaces, is attribute to a combination of factors, such as cavity selection and preparation, salivary contamination, restorative material, and the operator knowledge and clinical skills^2^ ^,^ ^28^. Particularly for approximal restorations, the difficulty in isolating the cervical area of cavities increases the risk of salivary or gingival fluid contamination and consequent microleakage, secondary caries formation and restoration failure^28^. Besides, large cavities did not show good survival results, probably because of bulk failures or pulpal effect^16^.

The present study showed no significant difference between GIC containing and not containing CHX, even after 1 year of treatment, confirming that the addition of chlorhexidine digluconate did not affect the mechanical properties of the restorative material. A recent study showed that the addition of 0.5% CHX to GIC improved antibacterial properties compared to conventional GIC, without affecting the clinical performance of class I restorations in young permanent molars until the 3-month follow-up. However, in contrast to our results, after 9 months the restoration success with GIC containing CHX (60%) was lower than the control group (85%)^14^. Differences of age, type of dentition and restorative material used could explain the disparities found in the studies. Although the oral hygiene index was not applied to this study participants, it is expected that a poor oral hygiene, since high scores of dfms were observed in a low age population (46.09±7.9 months), may have an overall impact on the survival of restoration^29^.

A significant reduction of mutans streptococci count on both saliva and biofilm from children at the 7^th^ day follow-up of ART procedure with GIC containing 1.25% CHX was observed in the present study, showing the antimicrobial action of CHX on buccal environment. The reduction in cariogenic microorganisms could be attributed to both cavity sealing and the antimicrobial properties of chlorhexidine digluconate. This antimicrobial agent has a wide spectrum of activity against Gram-positive bacteria, especially mutans streptococci^8^. However, the antibacterial effect of CHX associated with GIC seems to be limited, since after 3 months and one year of restoration, the experimental group did not show significant antibacterial effect in comparison to the control group. In a clinical trial study with a resin-modified glass ionomer cement liner containing chlorhexidine digluconate, it was found that the antibacterial action of the material on residual dentin lasts up to 90 days after the restorative procedure^5^. *In vivo* addition of 1% chlorhexidine diacetate to GIC showed comparable results to conventional GIC with regard to microleakage^21^. Differences in the selection of materials, sampling procedures and local of CHX action could explain the controversial results obtained by studies assessing antimicrobial efficacy of GIC containing CHX. In this study, we used a conventional high viscosity GIC that may easily release the antibacterial product while the resin-modified GIC may keep the same product for long time in the matrix, delaying its release. Furthermore, in this study, GIC was exposed to oral environment and it was subject to tooth abrasion that probably accelerated the chlorhexidine release.

The results of this study should be analyzed considering possible methodological limitations. One of them is the dropout rate, approximately 36%, that was higher than expected (20%), at one-year follow-up of the intervention. The main reasons for dropout were school transfer or traveling abroad with their parents. This fact could interfere in the reliability of results. Unfortunately, when the study was conducted, the schools have not been registered in the national education system yet. Nowadays, it is possible to find the schools in the national system. Another limitation is the combination of single and multiple-surface restorations for the statistical analysis, which difficults the comparison with other studies. The participation of younger children whose tooth restoration is considered more difficult and possible differences in the protocol of restorative treatment could also explain the low success rate of ART restorations in the present study. This low success rate raises the question about the longevity of approximal-ART restorations. Then, besides the antimicrobial effect, new restorative materials with enhanced mechanical properties could minimize cumulative effect of failures.

## Conclusions

The inclusion of CHX in GIC improves *in vitro* antimicrobial/antibiofilm action, without causing detrimental effects on cytotoxicity, mechanical and fluoride release properties of the material. Clinical follow-up demonstrated that ART restoration with GIC+CHX had a similar survival rate and better antimicrobial performance at the 7^th^ day when compared to conventional GIC. GIC containing chlorhexidine could be an alternative to traditional GIC indicated to ART, for it provides an additional antimicrobial effect that is interesting for children with high mutans streptococci counts during the initial adaptive phase of treatment.
